# Halloysite Nanotube-Ferrihydrite Incorporated Polyethersulfone Mixed Matrix Membrane: Effect of Nanocomposite Loading on the Antifouling Performance

**DOI:** 10.3390/polym13030441

**Published:** 2021-01-30

**Authors:** Syarifah Nazirah Wan Ikhsan, Norhaniza Yusof, Normi Izati Mat Nawi, Muhammad Roil Bilad, Norazanita Shamsuddin, Farhana Aziz, Ahmad Fauzi Ismail

**Affiliations:** 1 Advanced Membrane Technology Research Centre (AMTEC), N29A, Universiti Teknologi Malaysia, Johor Bahru 81310, Malaysia; syarifahnazirah.alyahya@gmail.com (S.N.W.I.); farhana@petroleum.utm.my (F.A.); afauzi@utm.my (A.F.I.); 2 School of Chemical and Energy Engineering, Faculty of Engineering, Universiti Teknologi Malaysia, Johor Bahru 81310, Malaysia; 3 Department of Chemical Engineering, Universiti Teknologi Petronas (UTP), Bandar Seri Iskandar 32610, Malaysia; normi_16000457@utp.edu.my (N.I.M.N.); mroil.bilad@utp.edu.my (M.R.B.); 4 Faculty of Integrated Technologies, Universiti Brunei Darussalam, Bandar Seri Begawan BE1410, Brunei; norazanita.shamsudin@ubd.edu.bn

**Keywords:** mixed matrix membrane, loading effect, halloysite nanotube, hydrous ferric oxide, nanocomposite

## Abstract

Membrane filtration is an attractive process in water and wastewater treatment, but largely restricted by membrane fouling. In this study, the membrane fouling issue is addressed by developing polyethersulfone (PES)-based mixed matrix membranes (MMMs) with the incorporation of hydrophilic nanoparticles as an additive. Ultrafiltration MMMs were successfully fabricated by incorporating different loadings of halloysite nanotube-ferrihydrates (HNT-HFO) into a polyethersulfone (PES) matrix and their performance was evaluated for the separation of bovine serum albumin (BSA) solution and oil/water emulsion. The results show that wettability is endowed to the membrane by introducing the additive aided by the presence of abundant -OH groups from the HFO. The loading of additive also leads to more heterogeneous surface morphology and higher pure water fluxes (516.33–640.82 L/m^2^h) more than twice that of the pristine membrane as reference (34.69 L/m^2^h) without affecting the rejection. The MMMs also provide much enhanced antifouling properties. The filtration results indicate that the flux recovery ratio of the modified membrane reached 100% by washing with only distilled water and a total flux recovery ratio of >98% ± 0.0471 for HNT-HFO-loaded membranes in comparison with 59% ± 0.0169 for pristine PES membrane.

## 1. Introduction

Membrane-based processes have played an important role in water and wastewater treatment. They are the main technology to address future water problems [1]. Despite the efficiency of the currently applied membrane materials, the occurrence of membrane fouling is still one of the impending factors that adversely affects the performance of the membrane over time. Fouling has prevented the reusability of membranes, as the performance was adversely affected due to the accumulation of foulants. Therefore, research to address the membrane fouling issue, particularly via membrane material developments, has been emerging. The modification of membranes has since been studied and one of the most promising modifications is through the development of mixed matrix membranes (MMMs). One of the goals of using this type of membrane was to incorporate the beneficial characteristics of various components in the membrane to achieve a better process performance. The prospect of being able to mix different materials, in particular nanomaterials, has opened up a plethora of possibilities for tailoring membrane properties. This includes the increased permeability or selectivity, decreased fouling and elimination of unique pollutants accomplished by integrating two or more processes or by developing an optimized filtration method [2]. It could give the physicochemical stability of one element, while offering the optimal morphology with increased hydrophilicity and enhanced resistance to fouling and mechanical, chemical and thermal stability over a broader pH and temperature spectrum [3,4,5,6].

Membrane surface hydrophobicity is one of the factors that leads to membrane fouling. It was found that improving membrane surface hydrophilicity may reduce membrane fouling to some degree [1]. Therefore, various approaches to hydrophilize the surface of the membrane have been investigated. The strategies include the mix of hydrophilic and hydrophobic polymer [7,8,9,10,11,12] and the deposition of hydrophilic films on hydrophobic material [13,14,15]. In general, the wettability of solid surfaces is regulated by the chemical compositions that specify the free energy of the surface and the surface morphology associated with the roughness. Furthermore, long-term flux reduction caused by irrecoverable fouling due to ineffective cleaning is a significant problem that needs to be addressed when developing an anti-fouling membrane material. The physicochemical interactions of the membrane and the solute in the feed solution could be influenced by a variety of membrane properties such as pore size, surface charge and hydrophilicity [16]. 

Polyethersulfone (PES) has been commonly used in membrane preparation due to considerable chemical and thermal resistances, large pH tolerances, fast handling, environmental endurance, and a wider range of pore sizes [17]. However, weak intrinsic antifouling properties limit its use and the chemical treatment regularly applied to address the issue eventually shortens the PES membrane lifespan [18]. Therefore, to enhance the antifouling properties of PES membranes, nanoparticles have been introduced, including the application of ferrihydrites (HFO) as an additive for PES membrane fabrication [19].

HFO is popular as an adsorbent material used for wastewater treatment, as it is inexpensive, easy to synthesize, ideal for both cation and anion sorption and often has a low chance of introducing another pollutant to the system [20]. The small size and high surface area of HFO nanoparticles make them an ideal adsorbent. The high surface area of HFO nanoparticles plays a significant role in the adsorption process [21]. 

Numerous authors have attempted to customize membrane development to enhance the antifouling performances for certain feeds, such as for oily wastewater treatment [22,23,24,25]. Faibish et al. [26] fabricated antifouling ceramic-supported polymer (CSP) ultrafiltration membranes by introducing hydrophilic poly(vinylpyrrolidone) (PVP) onto the surface of ceramic membrane supports for the treatment of synthetic microemulsions. It was found that the CSP membrane demonstrated a 50% improvement in the antifouling property and a higher oil rejection of over 20% compared to the native membrane used as a reference. Meanwhile, Chang et al. [27] modified commercial Al_2_O_3_ microfiltration membranes with nano-sized ZrO_2_ particles by in situ hydrolysis of ZrCl_4_ to separate an engine oil–water emulsion. It turned out that the modified coating enhanced the hydrophilicity of the membrane and contributed to reducing the membrane fouling of oil droplets by 35% with the addition of nanocomposite in the membrane. Rapid developments of functional materials with special wettability have offered a brand new avenue for developing advanced membrane materials by utilizing them as additives for performance boosting [2,28,29,30]. A more recent study has focused on the hydrophilic modification of PES membranes using graphene oxide and SiO_2_ by Alkindy et al. [31], in which the modified membrane recorded a high water flux of 2561 Lm^−2^h^−1^ and a 38% improvement of oil rejection in comparison with a pristine PES membrane. De Guzman et al. [32] have also employed hydrophilic nanoparticles to increase the antifouling performance of a cellulose acetate mixed matrix membrane by the incorporation of polydopamine-sulfobetaine methacrylate. An 8.85% increase in the flux recovery of oily wastewater was reported on the modified membrane, with a water flux of 583.64 Lm^−2^h^−1^.

Therefore, the addition of nanoparticles rich in oleophobic groups such as organo-silane as well as hydrophilic components has opened up the possibility to mitigate fouling problems in membranes. Therefore, in this study, we have explored the antifouling capabilities of a modified PES/HNT-HFO mixed matrix membrane (MMM). The effect of HNT-HFO loading on the resulting polyethersulfone (PES)-based membrane characteristics was first evaluated. Subsequently, the antifouling property induced by the HNT-HFO loading on developed MMMs was evaluated for the filtration of bovine serum albumin (BSA) and oil emulsion feeds. Finally, the fouling reversibility of the filtrations was also evaluated.

## 2. Materials and Methods 

### 2.1. Membrane Fabrication

The preparation of asymmetric flat sheet halloysite nanotube-ferrihydrates (HNT-HFO)-embedded PES membranes was conducted using the immersion precipitation phase inversion technique which was detailed in our previous work [33]. Exact amounts of HNT-HFO nanocomposite of 0.5, 1.0, 1.5 and 2.0 wt% (denoted as HH0.5, HH1.0, HH1.5 and HH2.0) was dissolved into methyl-2-pyrrolidone (NMP) which acted as the solvent. The notations of the prepared membranes are summarized in Table 1. Note that the membrane notation refers to the concentration of the HNT-HFO nanocomposite, i.e., HH1.0 was prepared from dope solution containing 1 wt% of HNT-HFO. Following the dispersion of HNT-HFO into the solvent, polyvinypyrollidone (PVP) (1.0 wt%) followed by PES (15 wt%) were dispersed into the mixture. The mixture was then continuously stirred for 24 h at ambient temperature using a magnetic stirrer. The PVP additive was soluble in both NMP and distilled water, and thus was expected to leach out from the membrane matrix during the phase inversion process [34]. The PVP leaching leads to the formation of macrovoids and thus producing highly porous membranes [35]. 

The homogeneous dope solution was poured onto a smooth glass plate and casted using a casting blade at a speed of 5 cms^−1^ to form a film under a wet casting thickness of 250 mm. Then, the cast film was immersed into a coagulation bath containing deionized water, together with the glass plate to allow phase inversion to take place. The solidified sheet of the resulting membrane was then transferred to another water bath after it peeled off naturally from the glass plate, indicating the completion of the phase inversion process. The membrane sheet was left in the water bath for 3 days to ensure complete removal of residual solvent and the PVP [36]. The membrane was then dried at ambient temperature (with humidity between 60 and 70%) before usage. 

### 2.2. Membrane Characterization

The morphology of the membrane surface was done using a field emission scanning electron microscope (FESEM, ZEISS SUPRA 35VP, Carl Zeiss, Jena, Germany). Prior to analysis, the membrane was freeze dried and coated with an ultra-thin layer of gold.

The membrane porosity was measured by the gravimetric method. The membrane sample was cut and wetted thoroughly using the deionized water. After fully wet, the water on the membrane surface was mopped, followed by weighing the wet membrane sample. The wet sample was then dried until a constant weight was obtained. The overall membrane porosity (*ε*, %) was calculated using the following Equation (1) [37].

(1) $$\varepsilon =\frac{\omega_{1}-\omega_{2}}{A\times l\times d_{w}}$$

where *ω*_1_ is the weight of the wet membrane (g); *ω*_2_ is the weight of the dry membrane (g); *A* is the membrane effective area (cm^2^), *d_w_* is the water density (0.998 g/cm^3^) and *l* is the membrane thickness (cm). The mean pore radius of the membrane, *r*, was determined using the Guerout–Elford–Ferry equation, as expressed in Equation (2).

(2) $$r=\sqrt{\frac{\left(2.9-1.75\varepsilon \right)\times 8\eta lQ}{\varepsilon \times A\times \Delta P}}$$

where *η* is the water viscosity at 25 °C, *l* is the membrane thickness (m), *Q* is the volume of permeate water per unit time (m^3^/s), A is the effective area of membrane (m^2^) and Δ*P* is the operating pressure (Pa). Membrane pore size (diameter) could be obtained by multiplying *r* by 2.

Additionally, the wettability of the membrane was also characterized via the contact angle (*θ*) measurement using the sessile drop method. The surface hydrophilicity is one of the significant properties of a membrane which affects both the membrane flux and the antifouling property. In addition, the contact angle (*θ*) may be used to elicit information regarding membrane surface energy for detailed interfacial analyses, as well as for qualitatively assessing the wettability, or hydrophobicity/hydrophilicity, of a membrane surface.

The membrane pore size distribution and average pore size distribution were evaluated based on SEM images using ImageJ software through a method reported by Pugazhenti and Kumar [1]. Five SEM pictures of each membrane were evaluated using the software in which the selected sections of the membranes were taken for evaluation. The area average pore diameter (*d_s_*) from SEM analysis of the membrane was evaluated by assuming the cylindrical porous texture of the membrane as:

$$d_{s}={\left[\frac{\sum_{i=1}^{n}n_{i}d_{i}^{2}}{\sum_{i=1}^{n}n_{i}}\right]}^{0.5}$$

where *n* is the number of pores, *d_i_* is the pore diameter (nm) of the *i*th pore.

### 2.3. Water Permeation Analysis

Pure water permeation flux (PWP) of the membranes was obtained using dead-end ultrafiltration (UF) using a method proposed by others [38]. Before the PWP test, the cut membrane with an area of 0.19 cm^2^ inside the cell was pressurized with distilled water at 101.32 kPa for 30 min for compaction purposes and the PWP test was done at 68.95 kPa with a constant feed rate of 50 L/h for 2 h. The PWP was determined using Equation (3):

(3) $$J_{w}=\frac{Q}{A\times \Delta T}$$

where *J_w_* is the pure water flux (L/m^2^/h), *Q* is the volume of permeate (in liters), *A* is the membrane area (m^2^) and Δ*T* is the sampling time (h).

### 2.4. Antifouling Test

The fouling propensity of the fabricated membranes was analyzed by using a method reported by Zhao et al. [39]. In this method, bovine serum albumin (BSA) as well as a synthetic crude oil emulsion were used as the feed for filtration. The synthetic oily wastewater was prepared using crude oil obtained from Terengganu Crude Oil Terminal (location: RE110), which is located offshore of the east of peninsular Malaysia. The characteristics of the crude oil are tabulated in Table 2.

The crude oil in water emulsion was prepared according to a method by Gohari et al., [2], in which crude oil of different concentrations, i.e., 10 and 1000 ppm, is mixed with deionized (DI) water under vigorous stirring at 350 rpm for about 30 min at room temperature. Once the process was completed, a solution with a uniform yellowish color was obtained. Considering the coalescence of oil droplets that may occur during a prolonged period of storage, synthetic wastewater was prepared a day before the experiment to keep the feed characteristics consistent. BSA solution was chosen as a protein foulant model for the study of membrane antifouling. On the other hand, the oil emulsion was used to evaluate the oil fouling propensity for applications of oily wastewater. After the PWP permeation test, the fabricated membranes were used for the filtration of 1.0 mg/mL of both oil and BSA solution. The pH of the BSA solution was kept at 7.0 by adjustments using 0.1 M phosphate-buffered solution.

Both the protein and the oil rejection rates were then calculated by determining the concentration of the BSA, the oil in the feed and the permeate solutions. The concentration was measured using a UV spectrophotometer (DR-5000, Hach, London, ON, Canada) at a wavelength of 278 nm. Both the BSA and oil rejection rates (R%) were calculated using Equation (4), in which *C_p_* and *C_f_* (mg/mL) are the protein concentrations of permeate and feed solutions, respectively.

(4) $$R\%=\left(1-\frac{C_{p}}{C_{f}}\right)\times 100\%$$

After foulant feed filtration, the membrane was then flushed using distilled water for approximately 15 min, followed by the PWP measurement of the cleaned membrane (*J_w_*_2_). The antifouling abilities of the membrane were found using the fouling recovery ratio (FRR) calculated using Equation (5).

(5) $$\mathrm{FRR}\%=\frac{J_{W2}}{J_{W1}}\times 100\%$$

where *Jw*_1_ is the PWP of the membrane and *Jw*_2_ is the repeated PWP after the fouling filtration test. The higher FRR value means that the membrane possessed better antifouling ability and higher washing efficiency [40].

To study the fouling process of the membrane in detail, several ratios were defined to describe the fouling-resistant property. The first ratio is defined in Equations (6)–(8).

(6) $$\mathrm{FRR}\%=1-\frac{J_{P}}{J_{W1}}\times 100\%$$

(7) $$rr\%=\frac{J_{w2-}J_{P}}{J_{W1}}\times 100\%$$

(8) $$rir\%=\frac{J_{w1-}J_{w2}}{J_{W1}}\times 100\%$$

where *J_p_* is the flux for feed solution and *rr* and *rir* are the degrees of flux loss caused by reversible fouling and irreversible fouling, respectively.

The antifouling resistance of the dry membrane against oil droplets was tested using a method used by Lai et al. [41]. The test was conducted by adding one single droplet of pure crude oil onto the surface of the dry membranes and then rinsed using 10 mL of water. The procedure was repeated for two cycles to ensure a clear visualization of the remaining oil stain could be observed on the dry membrane.

## 3. Results and Discussion

### 3.1. Membrane Wettability

Table 3 indicates that the PES neat membrane shows significant differences of contact angle value compared to the modified membranes. It can be inferred that the decrement of contact angle value (that corresponds to higher hydrophilicity/wettability) is due to the increasing of the HNT-HFO loadings. The surface hydrophilicity is one of the significant properties of the membranes, which can affect the flux and the antifouling ability of a membrane. The low water contact angle insinuates excellent hydrophilicity, which suggests that the embedding of HNT-HFO can further increase the hydrophilicity of the membranes. However, changes in the water permeability can be influenced by the type of nanofillers. For instance, the pristine PES membrane shows the highest contact angle of 81.39° and upon the incorporation of nanofillers, the contact angle decreases in line with the HNT-HFO loadings, in which HH2.0 shows the lowest contact angle of 50.30°. This is most likely due to the fact that HH2.0 has the highest adsorbed -OH, which led to higher water permeability. With a high number of hydroxyl groups that contribute to the water absorption rate and equilibrium water, HNTs yield a large surface region. Therefore, as the loading of the HNTs increases, the membrane hydrophilicity also increases, as also reported elsewhere [42].

**Figure 1:**
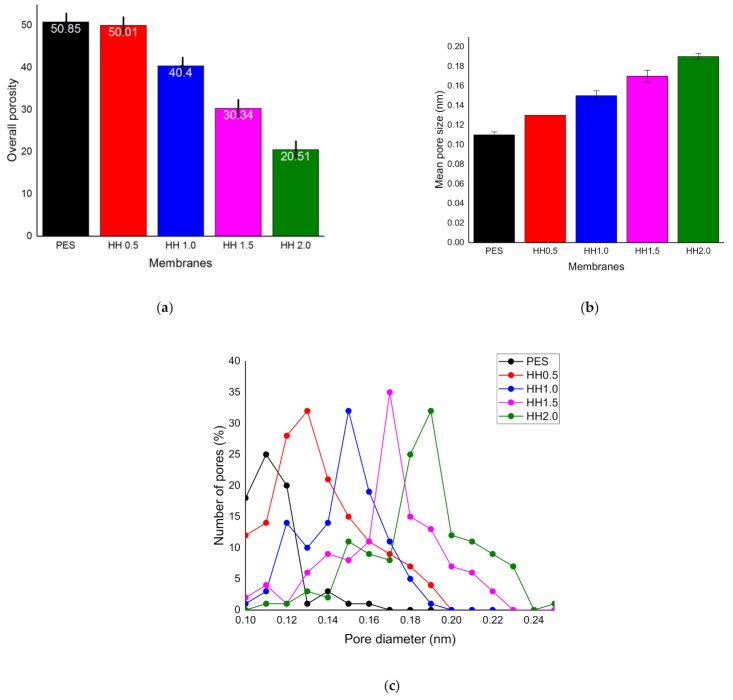
Membrane porosity analysis of the membrane, (**a**) overall porosity of mixed matrix membrane, (**b**) mean pore size of membrane, (**c**) pore size distribution.

Figure 1a shows that the membrane porosity decreases at higher loadings of the nanocomposite. The highest loading of the 2.0 wt% nanocomposite ratio resulted in 20.51% overall porosity, while pristine PES exhibited a mean porosity of 50.85%. In addition, the opposite trend was observed in the mean pore size of the membrane, in which the highest loading (HH2.0) showed the highest pore size of 0.19nm, while pristine PES showed a 0.11nm pore size, as depicted in Figure 1b. This difference can be physically observed in the SEM images. Figure 1c, on the other hand, depicts the pore size distribution, summarizing the evaluated values of percentage pore numbers with respect to different pore diameters. The pore size distribution can be closely correlated to the loading of nanoparticles in which, for all loading capacities, the maximum number of pores is in the range of 0.1 to 0.2 nm. In Figure 1b, the mean pore size decreases with the increase in nanoparticle loadings and the pore size distribution simultaneously narrows.

The decrease in porosity and pore size can be attributed to the delayed demixing mechanism in which the nanocomposite loading in the dope solution enhanced its viscosity. High solution viscosity hinders the exchange of solvent and nonsolvent during the phase inversion, which resulted in the formation of a less porous and denser membrane, as also detailed elsewhere [1]. Porosity and pore size can have a severe impact on the clean water PWP and flux decline during the actual filtration process. Low surface porosity can aggravate the effect of adsorption and fouling. This is due to a large build-up of solute near the pores [43].

**Figure 2:**
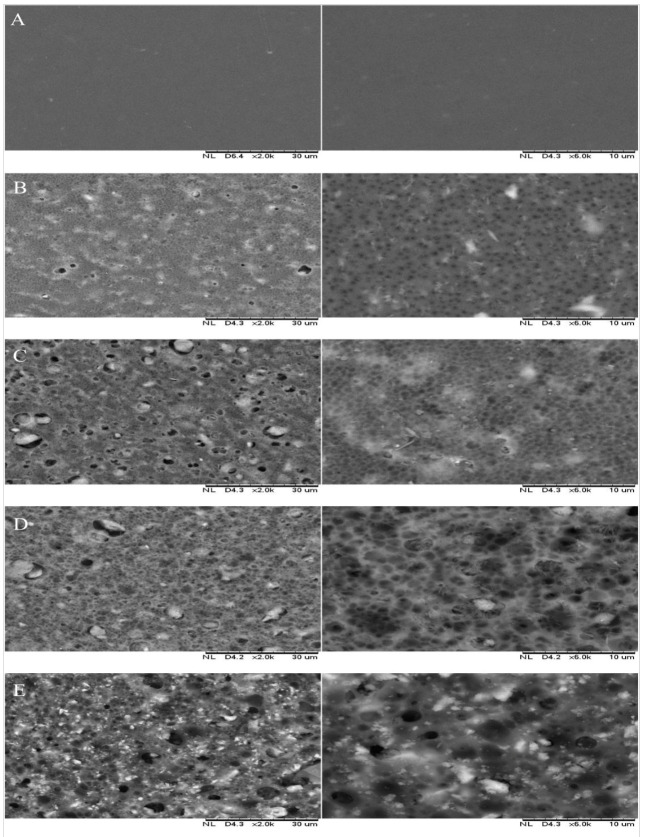
SEM surface images of (**A**) PES, (**B**) HH0.5, (**C**) HH1.0, (**D**) HH1.5 and (**E**) HH2.0 at 2000× and 6000× magnifications.

Figure 2 presents the SEM images of the membrane surfaces embedded with nanoparticles in comparison with the pristine PES membrane. The surface of the pristine membrane appeared to be smooth with only slight apparent surface pores, as opposed to those with nanoparticles and PVP as additives in which surface pores are clearly visible. This morphological characteristic of the modified membrane has contributed to its increased hydrophilic properties, as detailed elsewhere [44,45]. The increasing loading of HNT-HFO together with the presence of PVP affect the surface pore formation and the morphology in the modified membranes, as also reported elsewhere [45]. PVP acts as a pore former in the membrane [46]. Furthermore, the addition of nanocomposite has indirectly decreased the pore size of the modified membrane due to the addition of molecular chain lengths between bonds.

### 3.2. Water Permeation Analysis

**Figure 3:**
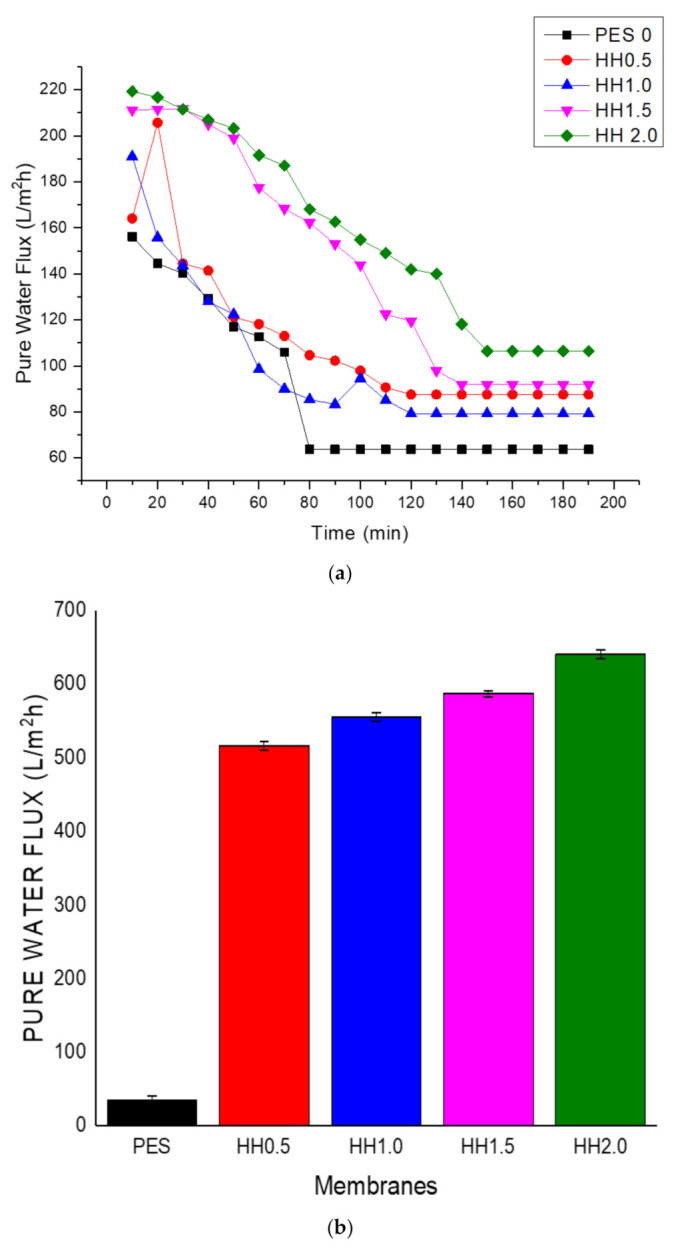
(**a**) Pure water flux (PWF) at different loadings of membrane over time, and (**b**) summary of the final PWFs.

Figure 3a shows the change in membrane flux over the filtration time. In the beginning, the permeation fluxes declined gradually up to a point at which the flux remained stable. As the permeation took place in the UF process, the fluxes of the modified membranes were always higher than the pristine PES, thanks to the improved hydrophilicity of the membrane surfaces. The increasing loading of the HNT-HFO enhances the PWP of the membrane at which HH1.5 and HH2.0 are much better than the PES and HH0.5. The steady state flux of the HH2.0 membrane is 640.82 L/m^2^h ± 0.0098, which is 1.71 times higher than that of the HH0.5 membrane (516.33 L/m^2^h ± 0.0027) and 10.1 times greater than the pristine PES membrane (34.69 L/m^2^h ± 0.0098). The loading of nanoparticles was only up to double the proportion of PES polymer (HH2.0 membrane) since further increases to the ratio would cause severe agglomeration which would make it untenable for membrane fabrication.

### 3.3. Antifouling Performance of the Membranes

**Figure 4:**
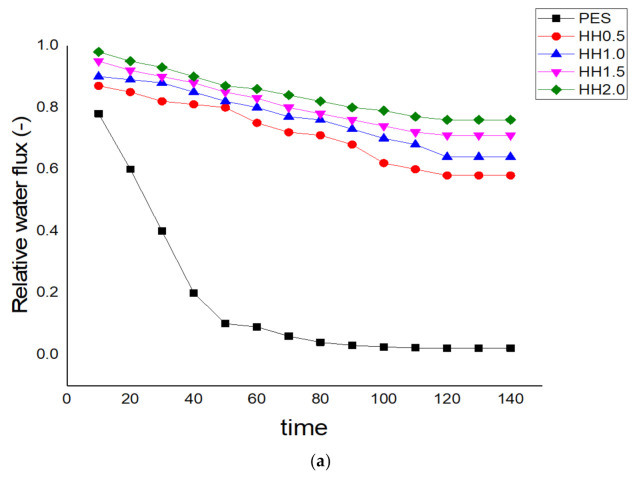

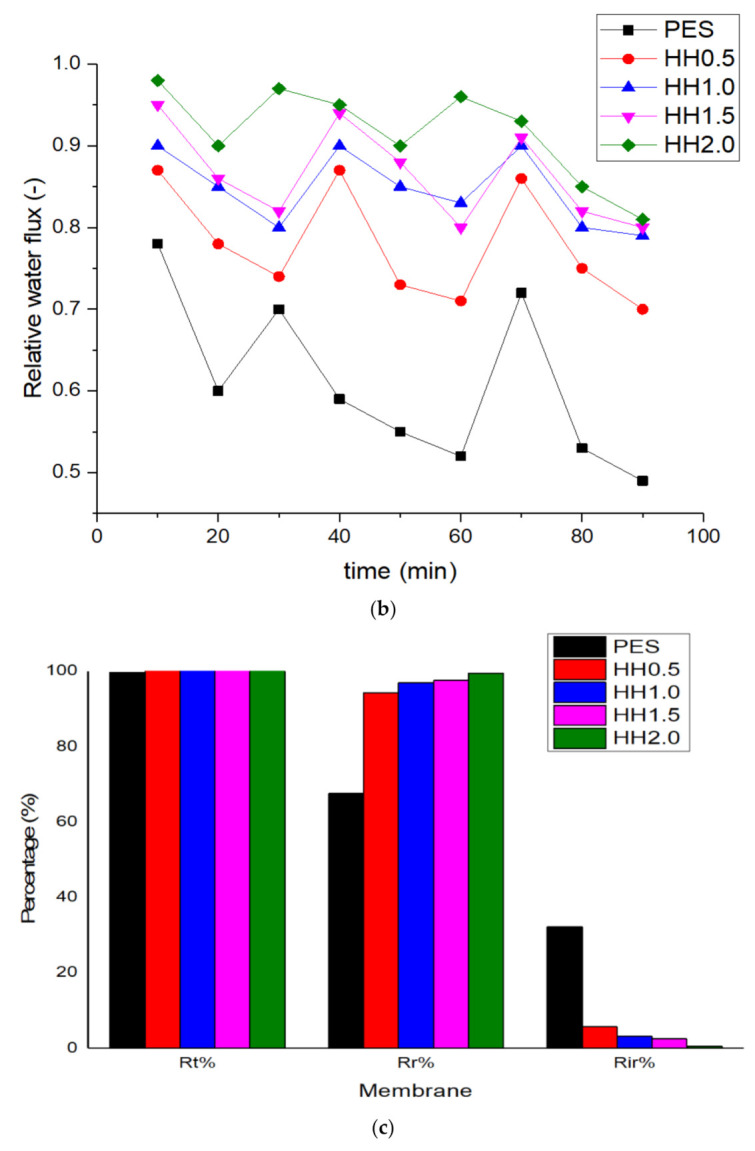
Time dependence of water permeation flux variations during membrane filtration of (**a**) bovine serum albumin (BSA) solution, (**b**) after washing with de-ionized water and (**c**) summary of corresponding fouling reversibility property.

The time-dependent, normalized permeation flux of the developed MMMs, as well as the pristine PES membranes treating the BSA solution, is shown in Figure 4a. After 30 min and 60 min of filtration of the BSA solution, the membranes were washed with deionized water, in which the water fluxes of the cleaned membranes were measured again and are shown in Figure 4b. In this study, the membranes incorporated with HNT-HFO yielded higher FRRs when compared to the pristine PES, mostly due to weak interactions between the foulant BSA and the membrane surface, as also reported by others [39]. Membrane fouling was caused by hydrophobic interactions between the foulants and the membranes.

The flux recovery performance of the membranes was evaluated with respect to several parameters, including flux recovery ratio, total flux decline ratio (*Rt*), reversible flux decline ratio (*Rr*) and irreversible flux decline ratio (*Rir*), as summarized in Figure 4c. The results show that the flux recovery ratio of the MMM is always 50% higher than the pristine membrane and the modified membrane showed a 20% better flux performance in comparison with the previous studies done on polyethersulfone membranes [31,47,48,49]. As the loading of nanocomposite increases, the flux recovery ratio of the membrane simultaneously increases, which proves that the addition of nanocomposite has improved the antifouling ability of the membrane. Similar results have been reported by previous researchers, for example, Zarghami et al. [3], who incorporated polydopamine as an additive in a PES membrane and the modified membrane showed up to a 79% improvement in the flux recovery [50]. Another study done by Alkindy et al. [4] has also reported a 38% flux recovery improvement for PES modified with GO/SiO_2_ nanoparticles [31]. In contrast with MMMs incorporated with HNT-HFO, the flux recovery of the highest loading has surpassed the percentage of those in the previous study, with the highest flux recovery percentage of 90%.

**Figure 5:**
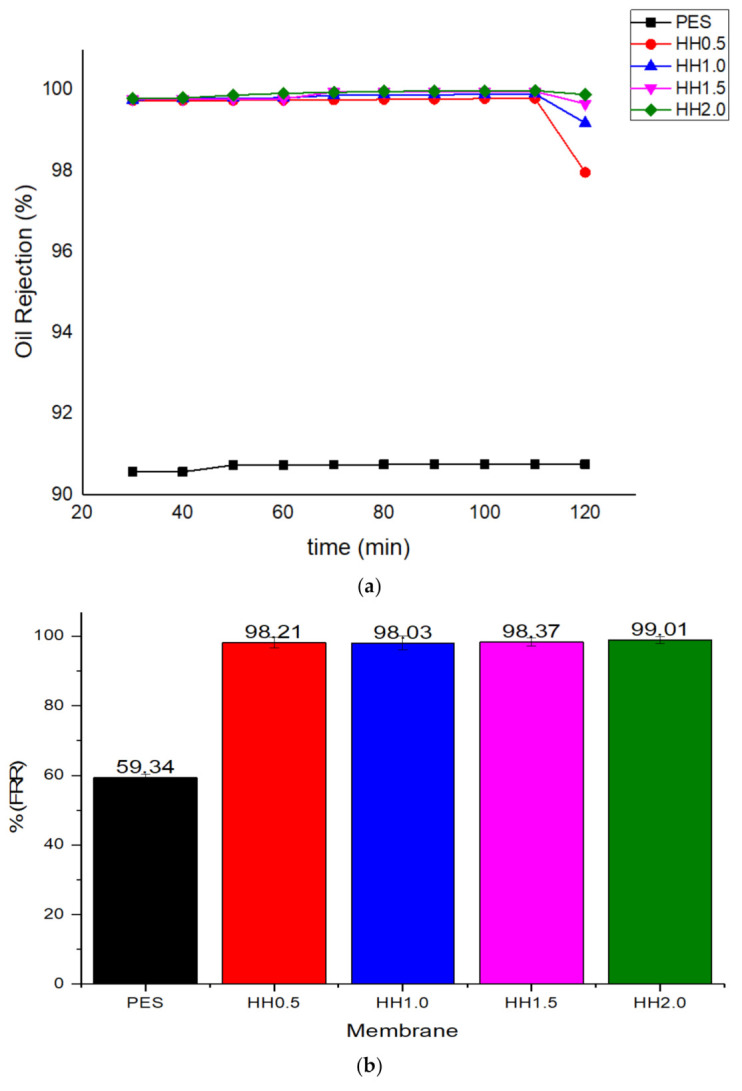
Oil rejection and fouling analysis of the membrane, (**a**) oil rejection percentage over time (%R) and (**b**) the flux recovery rate (FRR) of the pristine PES and the modified membranes.

The oil rejection percentage (%R) of the oily feed and FRR for the oily wastewater are shown in Figure 5. The modified membrane showed a high oil rejection percentage throughout the filtration process, with a 9% difference in comparison to the pristine PES. After 120 min, the percentage of oil rejection started to decrease, signifying that fouling had started. Differences in oil rejection can be seen at this time, in which HH2.0 showed the highest rejection of 99.89%, while HH0.5 showed a 97.96% rejection after 120 min of filtration. In Figure 5b, it can be observed that there are significant improvements of FRR in modified membranes when compared to pristine PES membrane and the membrane with the highest additive loading achieved the highest value of 99% ± 0.0027. The effect of HNT-HFO loading on fouling reduction was studied by assessing the properties and hydraulic performance of the modified membranes. It is worth mentioning that a low concentration of HNT-HFO contributed to low particle aggregation and thus reduced the nanoparticles’ effective surface area. Consequently, this condition results in lower FRR values due to the reduced surface hydrophilicity. Nonetheless, the amount of nanoparticle loading does not significantly affect the FRR. However, judging from the flux value, HH2.0 shows the best performance by showing the highest flux and almost complete fouling reversibility. A similar study done by previous researchers that employed HNT and Fe_2_O_3_ as additives in a polymer membrane has reported only up to 97% antifouling efficacy on the modified membrane [51,52,53,54].

**Figure 6:**
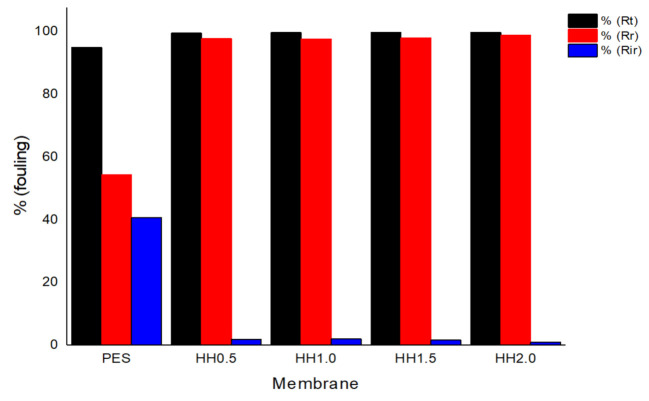
Comparison between total fouling and reversible and irreversible fouling.

Figure 6 shows that the pristine PES membrane has the lowest reversible fouling ratio but the highest irreversible fouling with comparable total fouling to other HNT-HFO blended membranes. The findings also suggest that the incorporation of HNT-HFO does not reduce the total fouling resistance (*R_t_*) and the loading amount does not significantly affect it. However, the main difference in membrane fouling resistance was observed in reversible and irreversible fouling. Reversible fouling increases for the modified membranes. Irreversible fouling also decreases by increasing the HNT-HFO content, showing an improvement of the membrane antifouling characteristic. The opposite behavior is seen for pristine PES due to the absence of the nanoparticles that otherwise could induce the antifouling property, which is in agreement with the study done by Ouda et al. [55]. Their study reported a 50% water flux reduction after oil feed permeation which is significantly higher than those reported in this study.

It is worth noting that the problem of irreversible membrane fouling is particularly significant in oil emulsion filtration. The irreversible decline in membrane performance is often caused by the strong physisorption and/or chemisorption of solutes onto the membrane surface and into its pores [56]. The flux decline, on the other hand, decreased with the increasing of nanocomposite, which in agreement with the trend of FRR. The cleaning of irreversibly fouled membranes is troublesome since it requires harsh chemicals or high-temperature thermal treatments [57]. Yet, due to the presence of irrecoverable fouling (not identified in this study), we still cannot fully recover the initial membrane performance [58]. Moreover, irreversible fouling could become more severe with each additional treatment and cleaning cycle due to the further adsorption of foulants Thus, it is essential to fabricate UF membranes from materials less susceptible to irreversible fouling. The modified membranes developed in this study show that the decline in the irreversible flux decreases, owing to the ability of the membrane to absorb water, and increases with the increasing of nanocomposite loading. The interaction between membrane material and oil changes, resulting in less permanent adsorption.

Fouling is governed by interactions between the foulant and the membrane surface [59]. Thus, the adhesion force of proteins against the surface of materials is an important parameter that allows the direct assessment of protein adsorption behavior at the interface [60]. It is also observed that the magnitude of the adhesion force correlates well with the fouling propensity of membranes and polymer-coated surfaces in the presence of organic foulant [61].

This study employed BSA and oil droplets in emulsion form as the fouling agent. The latter was applied to further test the pore fouling occurrence in the modified membrane because the PES adsorption capabilities still have the tendency to cause monolayer adsorption in the membrane pores. In addition to enhancing the water flux, the embedding of hydrophilic nanocomposites improves the PES-based membrane antifouling property against oil molecules. An outstanding antifouling property is very noteworthy as it can ensure the membrane reusability and lifespan and simplify the operation by decreasing the backwashing or cleaning frequencies [59]. Hence, in addition to comparing the total fouling rejection and irreversible fouling rate, this study has also employed a surface fouling test on the membrane.

Table 4 shows the membrane antifouling properties by adding a single oil drop and then rinsing with water twice. The test was done for the further depiction of the antifouling performance of the modified membranes by visualizing the effect of different loadings of nanocomposite via a physical antifouling resistance test. This test was performed by dropping concentrated crude oil onto the membrane surface. It can be clearly seen that the oil stain could be easily washed away from the membrane surface incorporated with hydrophilic nanofillers in comparison to the control pristine membrane, even with the use of a simple water rinsing process. The findings demonstrate that the improvement in membrane surface hydrophilicity not only enhanced membrane water flux, but also improved its fouling resistance by minimizing the deposition/adsorption of oil molecules.

## 4. Conclusions

The incorporation of HNT-HFO into PES-based membranes has been proven to enhance the hydraulic performance as well as anti-fouling property of the resulting modified membranes. The incorporation of higher loadings of HNT-HFO improves the membrane surface hydrophilicity, pure water flux and antifouling properties. Modified membrane permeation performance was improved significantly, with a PWP of 516.33–640.82 L/m^2^h in comparison to the pristine PES membrane as a reference, with a PWP of 34.69 L/m^2^h, without sacrificing its retention properties. All modified membranes showed excellent antifouling properties with FRRs of >98%, much higher than the FRR of the pristine PES (59%). The results of protein fouling tests via BSA filtration prove that the addition of nanocomposite has the ability to suppress the PES adsorption capability, which in turn avoids any pore fouling occurrence, hence increasing the hydrophilicity as well as selectivity of the modified membrane.

## Figures and Tables

**Table 1:** Composition of nanocomposite loadings of differently modified membranes.

Membrane Notation	Nanocomposite Loading (wt%)	Polyvinypyrollidone (PVP) Loading (wt%)
PES ^a^	0	1
HH0.5 ^b^	0.5	1
HH1.0 ^c^	1.0	1
HH1.5 ^d^	1.5	1
HH2.0 ^e^	2.0	1

^a^ Polyethersulfone; ^b^ HNT-HFO nanocomposite of 0.5 wt%; ^c^ HNT-HFO nanocomposite of 1.0 wt%; ^d^ HNT-HFO nanocomposite of 1.5 wt%; ^e^ HNT-HFO nanocomposite of 2.0 wt%.

**Table 2:** Characteristics of the petroleum crude oil used in this study.

Characteristics	Value
Viscosity	4.6 mm^2^s^−1^ at 20 °C
Refractive index	1.5
American Petroleum Institute (API) gravity	42.7
Sulfur content	0.044%

**Table 3:** Static contact angle of different loadings of membrane.

Membrane	Contact Angle
PES	81.39° ± 0.707
HH0.5	67.17° ± 0.728
HH1.0	63.40° ± 0.169
HH1.5	59.24° ± 0.141
HH2.0	50.30° ± 0.099

**Table 4:** Procedure to study the antifouling resistance of dry membranes against oil droplets.

Membrane	Drop of Oil	First Washing	Second Washing
PES	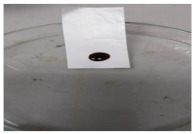	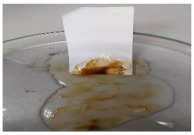	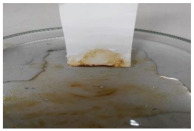
HH0.1	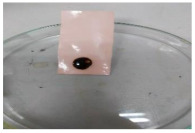	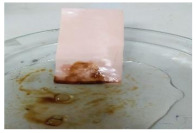	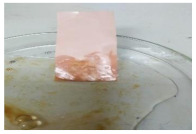
HH1.0	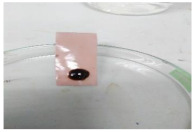	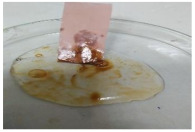	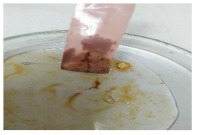
HH1.5	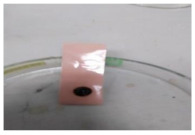	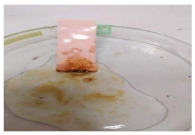	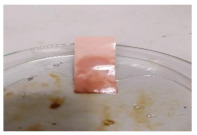
HH2.0	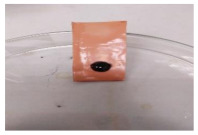	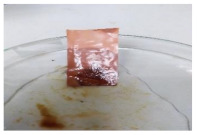	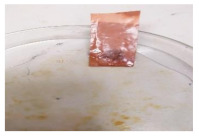

## Data Availability

The data presented in this study are available in within this article.
